# An Algorithm for Finding the Singleton Attractors and Pre-Images in Strong-Inhibition Boolean Networks

**DOI:** 10.1371/journal.pone.0166906

**Published:** 2016-11-18

**Authors:** Zhiwei He, Meng Zhan, Shuai Liu, Zebo Fang, Chenggui Yao

**Affiliations:** 1 Department of Mathematics, Shaoxing University, Shaoxing, China; 2 State Key Laboratory of Advanced Electromagnetic Engineering and Technology, School of Electrical and Electronic Engineering, Huazhong University of Science and Technology, Wuhan, China; 3 College of Science, Northwest A&F University, Yangling, China; 4 Department of Physics, Shaoxing University, Shaoxing, China; Semmelweis University, HUNGARY

## Abstract

The detection of the singleton attractors is of great significance for the systematic study of genetic regulatory network. In this paper, we design an algorithm to compute the singleton attractors and pre-images of the strong-inhibition Boolean networks which is a biophysically plausible gene model. Our algorithm can not only identify accurately the singleton attractors, but also find easily the pre-images of the network. Based on extensive computational experiments, we show that the computational time of the algorithm is proportional to the number of the singleton attractors, which indicates the algorithm has much advantage in finding the singleton attractors for the networks with high average degree and less inhibitory interactions. Our algorithm may shed light on understanding the function and structure of the strong-inhibition Boolean networks.

## Introduction

Revealing how a genetic regulatory network is organized for its function is one of the main topics in system biology [1–3]. With the contribution of experimental physiologists, many precise details of gene interactions as well as their functions have been revealed [3]. Based on the experimental data, different kinds of mathematical models have been developed, such as master equations [4], Monte-Carlo method [5, 6], stochastic model [7, 8], ordinary differential equations (ODE) [9–13], Boolean network method [14–16] and other mathematical models [17]. Among all the models, the Boolean network is a simple but powerful mathematical model. It was originally introduced by Kauffman [14] and has been quickly developed into many different types, including the general Boolean network model [15], the AND/OR Boolean network model [16], and the strong-inhibition Boolean network model [18], and so on. In the general Boolean network model, the most details of the network are taken into account. It can perfectly yield insight to global behavior of the network, however, it is difficult to analysis the general Boolean network due to the complexity of large genetic regulatory network. Further, a much simpler AND/OR Boolean network model where many details of the network are neglected is presented. Recently, a strong-inhibition Boolean network model which is a biophysically plausible gene model has been obtained from the transition of the general Boolean network model, as the inhibitory interactions often dominate the activations in some important genomic regulations [18]. The model has been successfully used to reveal the backbone motif structure of the cell-cycle network and reconstruct the network structure from evolution data [18–20], since the model has more details of the network, and a simple rule which is similar to the AND/OR Boolean network model. It is very suitable to be applied to study the genetic regulatory network.

To capture the biological meaning of the genetic regulatory network, it is very necessary to identify the stable states of the dynamic system, such as the cyclic attractor and the singleton attractor. Two different attractors correspond to different functional states of the network. As an example, the cyclic and singleton attractor correspond to cell growth and differentiated states (or apoptosis) in the cell-cycle network, respectively [21]. It is noteworthy that the identification of the attractors is of great importance and is very useful in many applications, such as the treatment of human cancers [22, 23], genetic engineering [24] and validate hypotheses on the transcription processes [25]. In order to identify all attractors of the genetic regulatory network, so far several methods have been proposed, including the methods relying on binary decision diagrams [26, 27], constraint programming [28], feedback vertex sets [29, 30], linear mapping [31]. Moreover, many of these methods have been developed to be more general and effective [32–34].

In particular, the identification of the singleton attractor is especially important for revealing the function of the genetic regulatory network [30]. To find the singleton attractors of the genetic regulatory network in the context of a Boolean network, several algorithms have been proposed. For instance, Leone et al. firstly applied the SAT (the satisfiability problem of Boolean formulas in conjunctive normal form) algorithms to identify the singleton attractor in a Boolean network [35]. Based on this observation, Tamura and Akutsu showed that the detection problem of singleton attractor for a Boolean network with maximum in-degree *k* can be reduced to (*k* + 1)-SAT, and presented an algorithm for detecting the singleton attractor of an AND/OR Boolean network in *O*(1.787^*N*^) time [36]. Subsequently, the authors and coworkers succeeded in improving the above algorithm, including proposed an *O*(1.587^*N*^) time algorithm for determining the singleton attractor of an AND/OR Boolean network [37], an *O*(1.757^*N*^) time algorithm for determining the singleton attractor of planar and nonplanar Boolean networks [38], the *O*(1.799^*N*^) and *O*(1.619^*N*^) time algorithm for determining the singleton attractor of Boolean networks with nested canalyzing functions and chain functions [39], respectively. In the *O*(1.799^*N*^) time algorithm, it was implicitly assumed that the network does not contain the positive self-loop. While allowing for the presence of positive self-loop, the singleton attractor detection problem was solved in *O*(1.871^*N*^) time [40]. Besides, Zhang et al. developed a quite fast algorithm using gene ordering [30]. It is shown that the algorithm can identify all singleton attractors for a random Boolean network with maximum indegree two with an average time *O*(1.19^*N*^). However, the average computational time complexity would increase (approximately *O*(1.5^*N*^) for maximum indegree ten) with increasing of maximum indegree. Recently, Zou proposed an algorithm by dividing the polynomial equation system into many subsystems [41]. Indeed, the problem of detection for the singleton attractor is still NP-hard [29, 30, 42]. Thus, it is not plausible to solve this problem efficiently in all cases. However, we can develop a new algorithm that is fast and can be applied in different mathematical models.

In this paper, we focus our attention on the strong-inhibition Boolean network model, and propose an algorithm for detecting the singleton attractors and pre-images of genetic regulatory network. Our algorithm is applied to the cell-cycle network of budding yeast, and can accurately identify all the singleton attractors of the network. Furthermore, we show that the average computational time increases exponentially with the growth of the network size *N*, and the order is approximately *O*(1.34^*N*^). Moreover, we discover that the computational time is proportional to the number of the singleton attractors of network. Based on this observation, we find that our algorithm has much advantage in finding the singleton attractors for the networks with high average degree and less inhibitory interactions. We further extend the algorithm for studying the pre-image problem. The pre-image problem (or predecessor problem), to find the set of all possible inputs that evolve into the given output, has been addressed by Wuensche in [43]. In recent years, several algorithms have been introduced, such as reverse algorithm [44], probabilistic algorithm [45]. But it has also been shown to be NP-hard in general [46]. In this paper, we extend the previous singleton attractor detection algorithm to find the pre-images of any given state. All pre-images and even the basin of one singleton attractor are successfully and accurately found.

The paper will be arranged as follows: in Sec. II, the state transition model is introduced. In Sec. III, the algorithm for finding the singleton attractor is given. In Sec. IV, we show an example for finding the singleton attractor with this algorithm, and the computational time of the algorithm for finding singleton attractors is analyzed. In Sec. V, we present the algorithm of finding the pre-images of any target network state. Finally, we give a summary in Sec VI.

## State transition model

Boolean network model is a discrete dynamical model of genetic regulatory networks. In this model, each node has only two states, 1 or 0, representing “on” (active) or “off” (inactive) state, respectively. For a network system with *N* components, the network state at time *t* is denoted by *S*(*t*) = (*S*_1_(*t*), *S*_2_(*t*), …, *S*_*N*_(*t*)). The network state in the next step is uniquely determined by the following rule [15]:

$$S_{i}\left(t+1\right)=\left\{\begin{array}{ccc} 1, & \;\text{if}\;\sum_{j=1}^{N}a_{ij}S_{j}\left(t\right)>0, & \\ 0, & \;\text{if}\;\sum_{j=1}^{N}a_{ij}S_{j}\left(t\right)<0, & \\ S_{i}\left(t\right), & \;\text{if}\;\sum_{j=1}^{N}a_{ij}S_{j}\left(t\right)=0, \end{array}\right.$$ (1)

where *i*, *j* = 1, 2, …, *N*. *A* = {*a*_*ij*_, *i*, *j* = 1, 2, …, *N*} is the adjacency matrix of the network, which denotes the interactions between all the components. *a*_*ij*_ can be positive, negative, or zero, standing for an activating interaction, inhibiting interaction or no interaction, respectively. Usually, *a*_*ii*_ take −1, 1, or 0, and *a*_*ij*_ take −*γ*, 1, or 0 for *j* ≠ *i*.

In fact, the inhibiting interactions are dominant for most biomolecular interactions, one prefers *γ* ≥ 1. Following the “dominant inhibition” assumption *γ* = ∞, which represents some typical biological transcriptional regulatory processes [47–49], the Eq (1) can be simplified into a logical equation [18]

$$S_{i}\left(t+1\right)=\left(\sum_{j\neq i}\left(S_{j}\left(t\right)g_{ij}\right)+S_{i}\left(t\right){\bar{r}}_{ii}+\bar{S_{i}\left(t\right)}g_{ii}\right)\prod_{j\neq i}\left(\bar{S_{j}\left(t\right)r_{ij}}\right),$$ (2)

where *r*_*ij*_ and *g*_*ij*_ represent the putative inhibitory and putative stimulatory edge from node *j* to *i*, respectively. The relation between *a*_*ij*_ and *g*_*ij*_, *r*_*ij*_ is

$$\left\{\begin{array}{ccccc} g_{ij}=1,\;r_{ij} & = & 0, & \;\;\text{if}\;a_{ij} & =1,\\ g_{ij}=0,\;r_{ij} & = & 1, & \;\;\text{if}\;a_{ij} & =-\gamma \;or\;-1,\\ g_{ij}=0,\;r_{ij} & = & 0, & \;\;\text{if}\;a_{ij} & =0, \end{array}\right.$$ (3)

where *i*, *j* = 1, 2, …, *N*. It should be noted that node *j* can’t have activating and inhibiting effect on node *i* at the same time, namely *g*_*ij*_ and *r*_*ij*_ can’t both take the value 1 for any fixed *i* and *j*. The addition, multiplication and bar in the Eq (2) represent the Boolean operator OR, AND, and NOT, respectively. This model is called strong-inhibition Boolean model due to the “dominant inhibition” assumption.

## The algorithm for finding the singleton attractor

To identify the singleton attractor which the network system will eventually evolve into a limited set of stable states, we should check all the states in the network state space. However, a great deal of time will be assumed for the large networks under this enumeration-based algorithm since the state space consists of 2^*N*^ different states. So it is very necessary to design more efficient method to identify the singleton attractor.

For the strong-inhibition Boolean model, the singleton attractors are solutions of the following equations

$$S_{i}\left(t\right)=\left(\sum_{j\neq i}\left(S_{j}\left(t\right)g_{ij}\right)+S_{i}\left(t\right){\bar{r}}_{ii}+\bar{S_{i}\left(t\right)}g_{ii}\right)\prod_{j\neq i}\left(\bar{S_{j}\left(t\right)r_{ij}}\right),$$ (4)

where *i*, *j* = 1, 2, …, *N*. We can obtain a concise equation by setting *S*_*i*_ = *S*_*i*_(*t*),

$$S_{i}=\left(\sum_{j\neq i}\left(S_{j}g_{ij}\right)+S_{i}{\bar{r}}_{ii}+\bar{S_{i}}g_{ii}\right)\prod_{j\neq i}\left(\bar{S_{j}r_{ij}}\right),\;\;for\;i=1,2,...,N.$$ (5)

Among all the interactions that regulate node *i*, we suppose that there are *h* positive interactions (*g*_*ij*_1__ = *g*_*ij*_2__ = … = *g*_*ij*_*h*__ = 1) and *m* negative interactions (*r*_*ik*_1__ = *r*_*ik*_2__ = … = *r*_*ik*_*m*__ = 1). Then Eq (5) can be written as:

$$S_{i}=\bar{S_{k_{1}}}\wedge \bar{S_{k_{2}}}\wedge \cdots \wedge \bar{S_{k_{m}}}\wedge \left(S_{j_{1}}\vee S_{j_{2}}\cdots S_{j_{h}}\vee X_{i}\right),$$ (6)

where ∧ and ∨ represent AND and OR, respectively. And *X*_*i*_ = 1 if *g*_*ii*_ = 1; *X*_*i*_ = 0 if *r*_*ii*_ = 1; *X*_*i*_ = *S*_*i*_ if *g*_*ii*_ = *r*_*ii*_ = 0. According to the definition in Ref. [40], one can see that the right side of Eq (6) is a chain function, which is a special case of nested canalyzing function (nc-function). It was proved by Akutsu et al. that finding a singleton attractor for an nc-network with chain functions remains NP-hard [39].

Then, some rules are gotten by analysing Eq (5),

**rule 1.** if *S*_*i*_ = 1 and *r*_*ji*_ = 1, then *S*_*j*_ = 0;
**rule 2.** if *S*_*i*_ = 1 and *r*_*ij*_ = 1, then *S*_*j*_ = 0;
**rule 3.** if *S*_*i*_ = 1, *r*_*ii*_ = 1, *g*_*ij*_0__ = 1 and ∑_*j* ≠ *j*_0__(*S*_*j*_ *g*_*ij*_) = 0, then *S*_*j*_0__ = 1;
**rule 4.** if *S*_*i*_ = 0 and ∑_*j* ≠ *i*_(*S*_*j*_ *r*_*ij*_) = 0, *g*_*ij*_0__ = 1 and ∑_*j* ≠ *j*_0__(*S*_*j*_ *g*_*ij*_) = 0, then *S*_*j*_0__ = 0;
**rule 5.** if *r*_*ii*_ = 1 and ∑_*j* ≠ *i*_(*S*_*j*_ *g*_*ij*_) = 0, then *S*_*i*_ = 0;
**rule 6.** if *g*_*ii*_ = 1 and ∑_*j* ≠ *i*_(*S*_*j*_ *r*_*ij*_) = 0, then *S*_*i*_ = 1;
**rule 7.** if ∑_*j* ≠ *i*_(*S*_*j*_ *r*_*ij*_) = 0, and there is a node *j*_0_ such that *S*_*j*_0__ *g*_*ij*_0__ = 1, then *S*_*i*_ = 1.

According to these rules and the given network structure, the states of other nodes can be determined if we already know the states of part of nodes. The states of its neighbors may be determined with the rules 1 − 4 if the state of a node is known; its state may be determined with the rules 5 − 7 if the state of a node is unknown. What’s more, according to the rules 1 and 2, we can find that if *S*_*i*_ = 1 and the node *i* has more putative inhibitory connections, the states of its neighbors can be easily determined. Based on these rules, we can design the following algorithm to identify the singleton attractors.

**Step 1.** Input the network matrix *A*, and a network state *S* = (*S*_1_, *S*_2_, …, *S*_*N*_), where the states of *l*_0_ (0 < *l*_0_ ≤ *N*) nodes are unknown while the states of other *N* − *l*_0_ nodes are known. Those *l*_0_ nodes are variable nodes as their states can be 1 or 0.
**Step 2.** Find the node *i*_0_ which has the most putative inhibitory connections among the variable nodes of the network.
**Step 3.** Let $S_{old}^{1}=S$ and assign *S*_*i*_0__ = 1. According to the rules 1 − 7, we can determine the states of more variable nodes, and obtain new network state *S*_*new*_. Throughout this process, a case may occur: according to one rule, the state of a variable node can be determined as 1, but it is determined as 0 with another rules. This contradiction means that the assignment of *S*_*i*_0__ = 1 is not appropriate, and the corresponding network state is not a singleton attractor of the network system, so we should remove it. On the other hand, if this case does not occur, we should remember the state *S*_*new*_ and count the number of its variable nodes *l*_*new*_. Moreover, if *l*_*new*_ > 0, we should remember this state and wait the next turn to begin **Step 1**; if *l*_*new*_ = 0, which means that the states of all nodes are determined and a new singleton attractor is found, return this singleton attractor.
**Step 4.** Let $S_{old}^{0}=S$ and assign *S*_*i*_0__ = 0. Do the same as those did in **Step 3**.

Next, we will prove that the states found with our algorithm are exactly all the singleton attractors of the network. In our algorithm, we determine the states of variable nodes according to the rules and remove the state if contradiction appears. Actually, the states found with our algorithm are states that do not contradict with the rules, here we use set *U* to denote them. And we use set *V* to represent all the singleton attractors of the network. Because a singleton attractor must satisfy Eq (5), so it obeys the rules, thus *V* is a subset of *U*, namely *V* ⊂ *U*. Afterwards, we will prove that *U* is also a subset of *V*.

Suppose that *U* is not a subset of *V*, there must be a state *S*^0^ such that *S*^0^ ∈ *U* and *S*^0^ ∉ *V*. Accordingly, the state *S*^0^ obeys all the rules but does not satisfy Eq (5). Then, there must be a node’s state, assuming $S_{i}^{0}$, such that

$$S_{i}^{0}\neq \left(\sum_{j\neq i}\left(S_{j}^{0}g_{ij}\right)+S_{i}^{0}{\bar{r}}_{ii}+\bar{S_{i}^{0}}g_{ii}\right)\prod_{j\neq i}\left(\bar{S_{j}^{0}r_{ij}}\right),$$ (7)

where $S_{i}^{0}=0$ or 1.

Firstly, assuming $S_{i}^{0}=0$ and inserting it into Eq (7), we obtain

$$\left(\sum_{j\neq i}\left(S_{j}^{0}g_{ij}\right)+g_{ii}\right)\prod_{j\neq i}\left(\bar{S_{j}^{0}r_{ij}}\right)=1.$$ (8)

Therefore, we get

$$\prod_{j\neq i}\left(\bar{S_{j}^{0}r_{ij}}\right)=1,$$ (9)

$$(\sum_{j\neq i}\left(S_{j}^{0}g_{ij}\right)+g_{ii})=1.$$ (10)

The Eq (9) indicates

$$\sum_{j\neq i}\left(S_{j}r_{ij}\right)=0.$$ (11)

And from Eq (10), we obtain

$$g_{ii}=1\;\;or\;\;\sum_{j\neq i}\left(S_{j}^{0}g_{ij}\right)=1.$$ (12)

Obviously, it contradicts with the rule 6 through Eq (11) and $S_{i}^{0}=0$ if *g*_*ii*_ = 1. And if $\sum_{j\neq i}\left(S_{j}^{0}g_{ij}\right)=1$, then there must be a node *j*_0_ such that $S_{j_{0}}^{0}g_{ij_{0}}=1$. So we can get

$$S_{j_{0}}^{0}=1\;\;and\;\;g_{ij_{0}}=1.$$ (13)

Through Eqs (11) and (13) and $S_{i}^{0}=0$, one can see that it contradicts with the rules 4 and 7. Therefore, it is impossible that *S*^0^ which obeys all the rules does not satisfy the Eq (5), if $S_{i}^{0}=0$.

Then, we assume $S_{i}^{0}=1$. Inserting it into Eq (7), we obtain

$$\left(\sum_{j\neq i}\left(S_{j}^{0}g_{ij}\right)+{\bar{r}}_{ii}\right)\prod_{j\neq i}\left(\bar{S_{j}^{0}r_{ij}}\right)=0.$$ (14)

Further, we get

$$\sum_{j\neq i}\left(S_{j}^{0}g_{ij}\right)+{\bar{r}}_{ii}=0\;\;\;or\;\;\;\prod_{j\neq i}\left(\bar{S_{j}^{0}r_{ij}}\right)=0.$$ (15)

If $\sum_{j\neq i}\left(S_{j}^{0}g_{ij}\right)+{\bar{r}}_{ii}=0$, then $\sum_{j\neq i}\left(S_{j}^{0}g_{ij}\right)=0$ and *r*_*ii*_ = 1. It contradicts with the rule 5 since $S_{i}^{0}=1$. If $\prod_{j\neq i}\left(\bar{S_{j}^{0}r_{ij}}\right)=0$, then there must be a node *j*_0_ such that

$$S_{j_{0}}^{0}=1\;and\;r_{ij_{0}}=1.$$ (16)

Through Eq (16) and $S_{i}^{0}=1$, we find that it contradicts with the rules 1 and 2. These results show that if $S_{i}^{0}=1$, it is also impossible that *S*^0^ which obeys all the rules does not satisfy Eq (5).

Now we can conclude that the state *S*^0^, which obeys all the rules but dissatisfies Eq (5), does not exist. It indicates that *U* is a subset of *V*, namely *U* ⊂ *V*. Therefore, *U* = *V* is proved. And we get the proof that the states found with our algorithm are exactly all the singleton attractors of the network. The code of this algorithm is provided in Supporting Information (S1 File).

## An example for finding the singleton attractor

**Fig 1:**
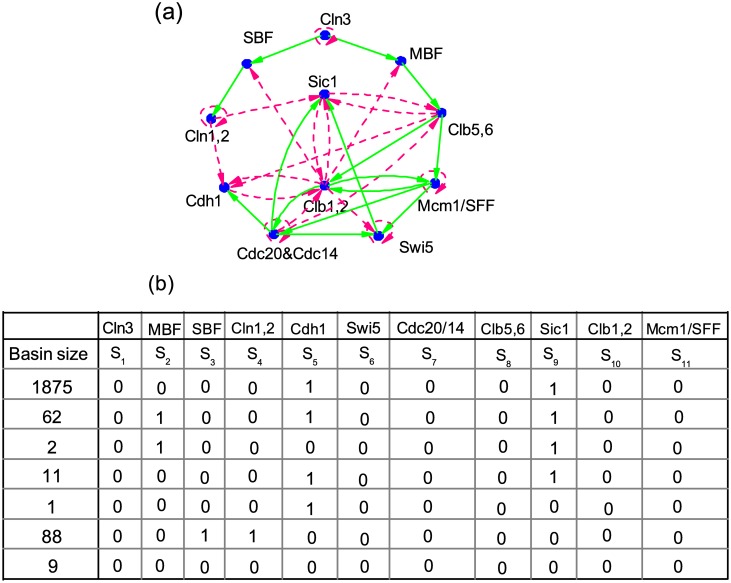
(a) The cell-cycle network of budding yeast. The green solid and pink dashed arrows represent positive and negative interactions, respectively. (b) Seven singleton attractors are found under the strong-inhibition Boolean model for the network in (a). The basin size for each singleton attractor is also given in the figure.

To verify the validity of the above algorithm, we apply it to detect the singleton attractors of the cell-cycle network for budding yeast which is well studied in the research of cell-cycle process [15]. As shown in Fig 1(a), the network consists of 11 nodes and 34 edges. According to prior works, there are 7 singleton attractors among 2^11^ = 2048 states in the state space of the cell-cycle network, as shown in Fig 1(b). Next, we will show how to find these singleton attractors with our algorithm.

**Fig 2:**
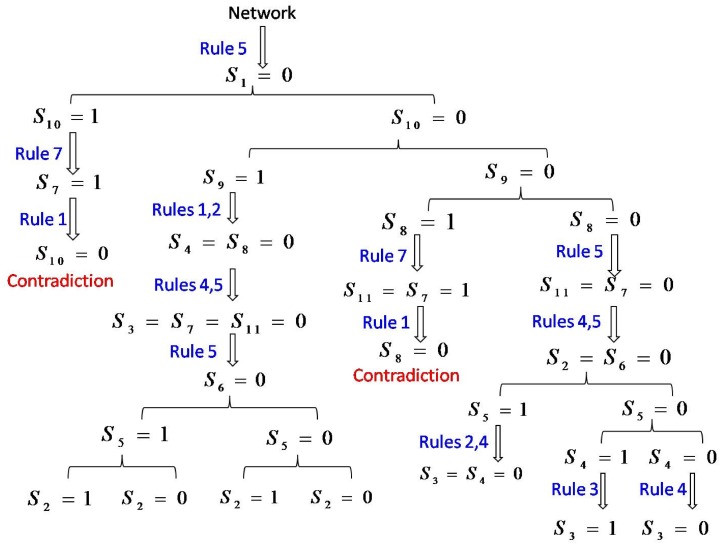
The flow chart for detecting all singleton attractors of the cell-cycle network of budding yeast.

A flow chart for detecting all the singleton attractors of the network is shown in Fig 2. We obtain *S*_1_ = 0 according to the known network structure and the rule 5. Next, we begin to detect the states of the variable nodes. We firstly rank all nodes from large to small according to the number of their putative inhibitory connections, the order is node 10, 9, 8, 5, 7, 4, 6, 3, 2, 11, 1.

Firstly, the network state (*S*_1_, *S*_2_, …, *S*_*N*_) is set to (0, *S*_2_, …, *S*_*N*_) with *l*_0_ = *N* − 1. Then, we get *S*_7_ = 1 according to the rule 7 by setting *S*_10_ = 1. Afterwards, *S*_10_ = 0 is obtained according to the rule 1. Now the contradiction appears, so the state *S* = (0, *S*_2_, …, *S*_9_, 1, *S*_11_) should be removed. On the other hand, assign *S*_10_ = 0. It turns out that no more nodes’ state can be determined, so we remember the state *S* = (0, *S*_2_, …, *S*_9_, 0, *S*_11_) with *l*_0_ = *N* − 2 and begin the next turn.

Secondly, we have network state *S* = (0, *S*_2_, …, *S*_9_, 0, *S*_11_) with *l*_0_ = *N* − 2. Then, we get *S*_8_ = 0 and *S*_4_ = 0 according to the rules 1 and 2 by assigning *S*_9_ = 1. We obtain *S*_3_ = 0 and *S*_7_ = *S*_11_ = 0 based on these known states and the rules 4, 5. *S*_6_ = 0 according to the rule 5. Now, no more nodes’ states can be determined, so we remember the state *S* = (0, *S*_2_, 0, 0, *S*_5_, 0, 0, 0, 1, 0, 0) with *l*_0_ = 2 and wait the next turn. Assign *S*_9_ = 0, we can not determine the states of any nodes, so we remember the state *S* = (0, *S*_2_, …, *S*_8_, 0, 0, *S*_11_) with *l*_0_ = 8 and begin the next turn.

Thirdly, there are two network states *S* = (0, *S*_2_, 0, 0, *S*_5_, 0, 0, 0, 1, 0, 0) with *l*_0_ = 2 and *S* = (0, *S*_2_, …, *S*_8_, 0, 0, *S*_11_) with *l*_0_ = 8. For the first network state, we find that all cases are ok through the assignment of *S*_2_ = 0 or 1 and *S*_5_ = 0 or 1. Therefore, we can obtain 4 singleton attractors: *S* = (0, 0, 0, 0, 1, 0, 0, 0, 1, 0, 0), (0, 1, 0, 0, 1, 0, 0, 0, 1, 0, 0), (0, 1, 0, 0, 0, 0, 0, 0, 1, 0, 0) and (0, 0, 0, 0, 0, 0, 0, 0, 1, 0, 0). For the second network state, assigning *S*_8_ = 1, we obtain *S*_11_ = 1, and then we obtain *S*_7_ = 1 according to the rule 7. We can get *S*_8_ = 0 according to *S*_7_ = 1 and the rule 1, when contradiction appears. So this state should be removed. On the other hand, we assign *S*_8_ = 0, according to the rule 5, we have *S*_11_ = 0, and further *S*_7_ = 0. Then according to the rules 4 and 5, we obtain *S*_2_ = 0 and *S*_6_ = 0, respectively. Now, we can not determine the states of any nodes, so we remember the state *S* = (0, 0, *S*_3_, *S*_4_, *S*_5_, 0, 0, 0, 0, 0, 0) with *l*_0_ = 3 and begin the next turn.

Fourthly, the network state is *S* = (0, 0, *S*_3_, *S*_4_, *S*_5_, 0, 0, 0, 0, 0, 0) with *l*_0_ = 3. We assign *S*_5_ = 1, *S*_4_ = 0 is determined according to the rule 2. Further, we have *S*_3_ = 0 according to the rule 4. Therefore, we can return the singleton attractor *S* = (0, 0, 0, 0, 1, 0, 0, 0, 0, 0, 0). On the other hand, we assign *S*_5_ = 0, and find that the states of nodes 3 and 4 can not be determined. So we remember the state *S* = (0, 0, *S*_3_, *S*_4_, 0, 0, 0, 0, 0, 0, 0) with *l*_0_ = 2 and begin the next turn.

Fifthly, the network state *S* = (0, 0, *S*_3_, *S*_4_, 0, 0, 0, 0, 0, 0, 0) with *l*_0_ = 2 is gotten. Assigning *S*_4_ = 1, we have *S*_3_ = 1 according to the rule 3. So we return the singleton attractor *S* = (0, 0, 1, 1, 0, 0, 0, 0, 0, 0, 0). Next, we assign *S*_4_ = 0, we obtain *S*_3_ = 0 according to the rule 4. So we return the singleton attractor *S* = (0, 0, 0, 0, 0, 0, 0, 0, 0, 0, 0).

Finally, we get all the singleton attractors: *S* = (0, 0, 0, 0, 1, 0, 0, 0, 1, 0, 0), (0, 1, 0, 0, 1, 0, 0, 0, 1, 0, 0), (0, 1, 0, 0, 0, 0, 0, 0, 1, 0, 0), (0, 0, 0, 0, 0, 0, 0, 0, 1, 0, 0), (0, 0, 0, 0, 1, 0, 0, 0, 0, 0, 0), (0, 0, 1, 1, 0, 0, 0, 0, 0, 0, 0) and (0, 0, 0, 0, 0, 0, 0, 0, 0, 0, 0). These seven states are exactly all the singleton attractors of the cell-cycle network of budding yeast as shown in Fig 1(b).

Furthermore, the singleton attractors of five classical networks are detected. As shown in Table 1, the number of singleton attractors (*NS*) and running time of our algorithm for each network are given in columns 4 and 5. We also give the results of the enumeration-based algorithm in columns 6 and 7. Comparing our algorithm with the enumeration-based algorithm, our algorithm can not only find the same number of the singleton attractors with the enumeration-based algorithm (columns 4 and 6), but also decrease extremely the running time (columns 5 and 7). Especially for the largest network (T-cell receptor), it took round 0.0468 seconds for our algorithm, whereas the enumeration-based algorithm spent more than 40 hours.

**Table 1:** Experiment results of five classical networks.

	Network size		Our algorithm		Enum. algorithm	
Network name	Nodes	Edges	*NS*	time(*s*)	*NS*	time(*s*)
Cancer cell [50]	8	21	2	<10^−8^	2	<10^−8^
Budding yeast [16]	11	34	7	<10^−8^	34	<10^−8^
Arabidopsis thaliana [51]	15	43	188	<10^−8^	43	0.0156
T-helper cell [52]	23	35	4453	0.1404	4453	2.5896
T-cell receptor [53]	40	596	1364	0.0468	1364	147080.09

Next, we will show the computational time of our algorithm by numerical simulations on random gene regulatory networks. Similar to the ER random network [54], there exits interaction (*a*_*ij*_ ≠ 0) from node *j* to *i* (*i* = *j* is allowed) with the probability *p*. Usually, the parameter *p* is determined by *p* = 〈*k*〉/*N*, where 〈*k*〉 is the average degree of the network. Furthermore, *a*_*ij*_ ≠ 0 has been assumed to take an independent value (±1) distributed according to a two-point probability distribution function. More specifically, we adopt the following values at random:

$$a_{ij}=\left\{\begin{array}{cc} -1,\; & \;\text{the inhibiting interaction with probability }r,\\ 1, & \;\text{the activating interaction with probability }1-r. \end{array}\right.$$ (17)

**Fig 3:**
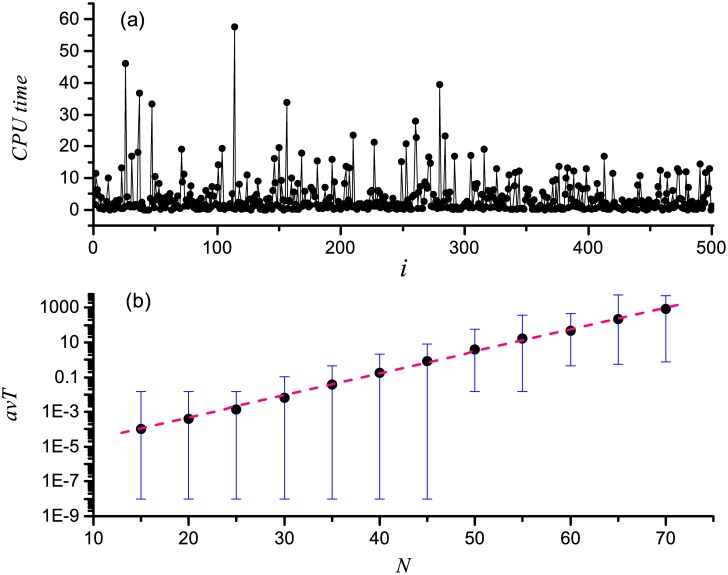
(a) The CPU computational time assumed of the algorithm for finding the singleton attractors of *M* (*M* = 500) random genetic regulatory network with *N* = 50, 〈*k*〉 = 3, *r* = 0.4. (b) Semi-logarithmic plot of the averaged CPU computational time (*avT*) as a function of the network size *N*. For each *N*, the value of *avT* is averaged over *M* (*M* ≥ 500) samples. And the error bars denote the range of CPU computational time, while the upper and lower ends of bars represent the maximum and minimum values, respectively. The straight dashed line is linearly fit of the data, indicative of the correlation *avT* ∝ 1.34^*N*^.

The average degree is about 2 ∼ 4 for many biological networks, and the amount of inhibiting interactions is less than that of activating interactions [15, 55–57], so 〈*k*〉 = 3 and *r* = 0.4 are fixed. We set *N* = 50 and generate 500 sample networks. The CPU computational time for finding all the singleton attractors of each network with above algorithm is calculated and shown in Fig 3(a). As we can see, the singleton attractors of every network can be detected, and the time is no more than 1 minute. This indicates that the singleton attractors of a network can be found efficiently with our algorithm.

The average CPU computational time *avT* as a function of the network size *N* is also computed. The result is shown in a semi-logarithmic plot in Fig 3(b), with a straight fit (dashed line) to guide an eye. The value of *avT* for each *N* is averaged over 500 samples. And the error bars denote the range of CPU computational time, where the upper and lower ends of bars represent the maximum and minimum values, respectively. In the figure, the average CPU computational time increases as the growth of network size *N*. This behavior is well characterized by the exponential relationship, *avT* ∝ 1.34^*N*^. This exponential increase also exists between the maximum CPU computational time and the size of network.

**Fig 4:**
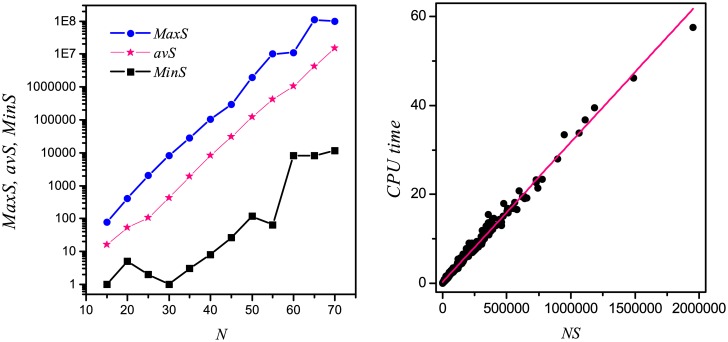
(a) Semi-logarithmic plot of the maximum (*MaxS*), average (*avS*), and minimum (*MinS*) number of the singleton attractors of networks as the function of the size of system *N*. (b) Semi-logarithmic plot of the CPU computational time vs the number of the singleton attractors (*NS*). The straight line is linearly fit of the data to guide an eye.

Although the average and maximum CPU computational time increase exponentially with the increase of *N*, we do not know why the minimum CPU computational time is very short for each *N*, as shown in Fig 3(b). To understand this question, the maximum, average, and minimum number of the singleton attractors as the function of the size of system *N* are shown in Fig 4(a), respectively. From this figure, we find that the average and the maximum number of the singleton attractors increase exponentially with *N* increasing, and there is not exponential relation between the minimum number of the singleton attractors and the size of system. Furthermore, the sample networks with size of *N* = 50 are generated. We plot the CPU computational time against the number of singleton attractors (*NS*) of the sample networks, and show the result in Fig 4(b). We find that the computational time increases linearly with the number of the singleton attractors increasing. Based on these observations, the minimum CPU computational time nonexponentially increases with the size of system, as the exponential relation between the minimum number of the singleton attractors and the size of system does not exist.

**Fig 5:**
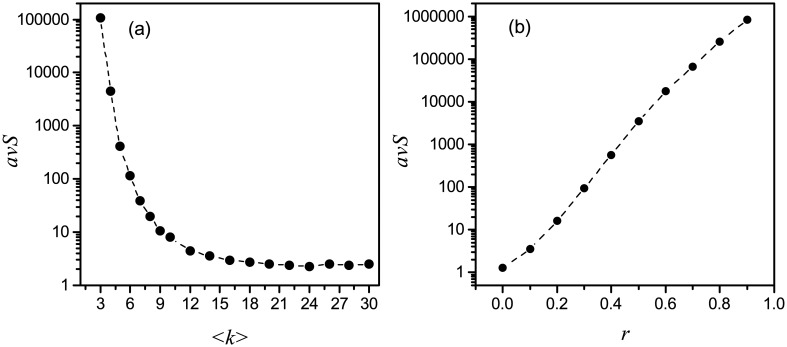
(a) Semi-logarithmic plot of the *avS* as a function of the average degree 〈*k*〉 for networks with *N* = 50, *r* = 0.4. (b) Semi-logarithmic plot of the *avS* against the inhibiting interactions probability *r* for networks with *N* = 50, 〈*k*〉 = 5. Every data in the plots is averaged over *M* (*M* ≥ 500) samples.

Following the above observation, we may want to know what characteristics of networks could have small number of singleton attractors. So we calculate the average number of singleton attractors of networks for different average degree 〈*k*〉′*s* and inhibiting interactions probability *r*′*s*. In Fig 5(a) and 5(b), we show the semi-logarithmic plots of *avS* (the average number of singleton attractors) as a function of 〈*k*〉 and *r*, respectively. We can find that *avS* decreases extremely fast with the increase of 〈*k*〉 and increases exponentially with the increase of *r*. According to the above observation, these results indicate that less computational time is assumed to find the singleton attractors of the networks with high average degree and less inhibitory interactions.

## Finding the pre-images of any target network state

The pre-images are the set of all possible inputs which evolve into a given output. For the strong-inhibition Boolean model, the pre-images of a given state *S*^0^(*t* + 1) (target state) are the solutions of

$$S_{i}^{0}\left(t+1\right)=\left(\sum_{j\neq i}\left(S_{j}\left(t\right)g_{ij}\right)+S_{i}\left(t\right){\bar{r}}_{ii}+\bar{S_{i}\left(t\right)}g_{ii}\right)\prod_{j\neq i}\left(\bar{S_{j}\left(t\right)r_{ij}}\right),$$ (18)

For simplity, we will use *S*_*j*_ to represent *S*_*j*_(*t*). Through analysing this equation, we can still obtain the following rules if $S_{i}^{0}\left(t+1\right)=1$,

**rule 1.1.** if *r*_*ij*_ = 1, then *S*_*j*_ = 0;
**rule 1.2.** if *r*_*ii*_ = 1, *g*_*ij*_0__ = 1, ∑_*j* ≠ *i*_(*S*_*j*_*r*_*ij*_) = 0 and ∑_*j* ≠ *j*_0__(*S*_*j*_*g*_*ij*_) = 0, then *S*_*j*_0__ = 1;
**rule 1.3.** if *g*_*ii*_ = 0, *r*_*ii*_ = 0, ∑_*j* ≠ *i*_(*S*_*j*_*r*_*ij*_) = 0 and ∑_*j* ≠ *i*_(*S*_*j*_*g*_*ij*_) = 0, then *S*_*i*_ = 1;
**rule 1.4.** if *g*_*ii*_ = 0, *g*_*ij*_0__ = 1, ∑_*j* ≠ *i*_(*S*_*j*_*r*_*ij*_) = 0, ∑_*j* ≠ *j*_0__(*S*_*j*_*g*_*ij*_) = 0 and *S*_*i*_ = 0, then *S*_*j*_0__ = 1;
**rule 1.5.** if *r*_*ii*_ = 1 and ∑_*j* ≠ *i*_(*S*_*j*_*g*_*ij*_) = 0, then contradiction appears.
Some rules are given if $S_{i}^{0}\left(t+1\right)=0$,
**rule 2.1.** if ∑_*j* ≠ *i*_(*S*_*j*_*r*_*ij*_) = 0 and there is a node *j*_0_ such that *g*_*ij*_0__ = 1, then *S*_*j*_0__ = 0;
**rule 2.2.** if *g*_*ii*_ = 0, *r*_*ii*_ = 0 and ∑_*j* ≠ *i*_(*S*_*j*_*r*_*ij*_) = 0, then *S*_*i*_ = 0;
**rule 2.3.** if *g*_*ii*_ = 1, *r*_*ij*_0__ = 1, and ∑_*j* ≠ *j*_0__(*S*_*j*_*r*_*ij*_) = 0, then *S*_*j*_0__ = 1;
**rule 2.4.** if *r*_*ij*_0__ = 1, ∑_*j* ≠ *i*, *j* ≠ *j*_0__(*S*_*j*_*r*_*ij*_) = 0 and *g*_*ii*_ = 1 or there is a node $j_{0}^{{}^{\prime }}$ such that $S_{j_{0}^{{}^{\prime }}}g_{ij_{0}^{{}^{\prime }}}=1$, then *S*_*j*_0__ = 1;
**rule 2.5.** if *g*_*ii*_ = 1 and ∑_*j* ≠ *i*_(*S*_*j*_*r*_*ij*_) = 0, then contradiction appears.

If one observes carefully those rules, we find that the previous algorithm for detecting the singleton attractors can be extended to find the pre-images for any target network state. Here, we just need to input the target state in **Step 1** and replace the rules 1 − 7 with rules 1.1 − 1.5 and 2.1 − 2.5 in **Steps 3** and 4.

Next, we will prove that the states found with this algorithm are precisely all the pre-images of the target network state. Here, we still use *U* and *V* to stand for the set of the states found with this algorithm and the set of all the pre-images of the target network state, respectively. Obviously, any pre-image of *S*^0^(*t* + 1) must satisfy Eq (18), and it follows certainly all the rules. So *V* is a subset of *U*, namely *V* ⊂ *U*. Therefore, to prove *U* = *V*, we just need to prove *U* ⊂ *V*.

Suppose that *U* is not a subset of *V*, then there must be a state *S*^0^(*t*) such that *S*^0^(*t*) ∈ *U* and *S*^0^(*t*)∉*V*. The state *S*^0^(*t*) obeys all the rules but does not satisfy Eq (18), namely, there is a node’s state which dissatisfies the equation. We assume that this state is $S_{i}^{0}\left(t+1\right)$, and we have

$$S_{i}^{0}\left(t+1\right)\neq \left(\sum_{j\neq i}\left(S_{j}^{0}\left(t\right)g_{ij}\right)+S_{i}^{0}\left(t\right){\bar{r}}_{ii}+\bar{S_{i}^{0}\left(t\right)}g_{ii}\right)\prod_{j\neq i}\left(\bar{S_{j}^{0}\left(t\right)r_{ij}}\right),$$ (19)

where $S_{i}^{0}\left(t+1\right)=0$ or 1.

Firstly, assuming $S_{i}^{0}\left(t+1\right)=1$ and inserting it into Eq (19), we obtain

$$\left(\sum_{j\neq i}\left(S_{j}^{0}\left(t\right)g_{ij}\right)+S_{i}^{0}\left(t\right){\bar{r}}_{ii}+\bar{S_{i}^{0}\left(t\right)}g_{ii}\right)\prod_{j\neq i}\left(\bar{S_{j}^{0}\left(t\right)r_{ij}}\right)=0.$$ (20)

This equation indicates

$$\prod_{j\neq i}\left(\bar{S_{j}^{0}\left(t\right)r_{ij}}\right)=0$$ (21)

or

$$\sum_{j\neq i}\left(S_{j}^{0}\left(t\right)g_{ij}\right)+S_{i}^{0}\left(t\right){\bar{r}}_{ii}+\bar{S_{i}^{0}\left(t\right)}g_{ii}=0.$$ (22)

Through Eq (21), there must be a node *j*_0_ such that $S_{j_{0}}^{0}\left(t\right)=1$ and *r*_*ij*_0__ = 1, we can find that it contradicts with the rules 1.1. So it is necessary that $\prod_{j\neq i}\left(\bar{S_{j}^{0}\left(t\right)r_{ij}}\right)=1$, hence we have ∑_*j* ≠ *i*_(*S*_*j*_*r*_*ij*_) = 0. If Eq (22) holds, one case is $\sum_{j\neq i}\left(S_{j}^{0}\left(t\right)g_{ij}\right)=0$, *g*_*ii*_ = *r*_*ii*_ = 0 and $S_{i}^{0}\left(t\right)=0$, it contradicts with the rule 1.3 through ∑_*j* ≠ *i*_(*S*_*j*_*r*_*ij*_) = 0; the other case is $\sum_{j\neq i}\left(S_{j}^{0}\left(t\right)g_{ij}\right)=0$ and *r*_*ii*_ = 1, we can observe that the contradiction appears according to the rule 1.5. Therefore, it is impossible that *S*^0^(*t*) which obeys all the rules does not satisfy Eq (18), if $S_{i}^{0}\left(t+1\right)=1$.

Afterwards, we assume $S_{i}^{0}\left(t+1\right)=0$. Inserting it into Eq (19), we obtain

$$\left(\sum_{j\neq i}\left(S_{j}^{0}\left(t\right)g_{ij}\right)+S_{i}^{0}\left(t\right){\bar{r}}_{ii}+\bar{S_{i}^{0}\left(t\right)}g_{ii}\right)\prod_{j\neq i}\left(\bar{S_{j}^{0}\left(t\right)r_{ij}}\right)=1.$$ (23)

From this equation, we can get

$$\prod_{j\neq i}\left(\bar{S_{j}^{0}\left(t\right)r_{ij}}\right)=1,$$ (24)

$$(\sum_{j\neq i}\left(S_{j}^{0}\left(t\right)g_{ij}\right)+S_{i}^{0}\left(t\right){\bar{r}}_{ii}+\bar{S_{i}^{0}\left(t\right)}g_{ii})=1.$$ (25)

The Eq (24) indicates

$$\sum_{j\neq i}\left(S_{j}^{0}\left(t\right)r_{ij}\right)=0.$$ (26)

And from Eq (25), we obtain

$$\sum_{j\neq i}\left(S_{j}^{0}\left(t\right)g_{ij}\right)=1$$ (27)

or

$$S_{i}^{0}\left(t\right){\bar{r}}_{ii}+\bar{S_{i}^{0}\left(t\right)}g_{ii}=1.$$ (28)

If Eq (27) works, then there must be a node *j*_0_ such that

$$S_{j_{0}}^{0}\left(t\right)=1\;\;and\;\;g_{ij_{0}}\left(t\right)=1.$$ (29)

Through Eqs (26) and (29), we can find that it contradicts with the rule 2.1. On the other hand, if Eq (28) is valid, two cases should be discussed: one case is *g*_*ii*_ = *r*_*ii*_ = 0 and $S_{i}^{0}\left(t\right)=1$, it contradicts with the rule 2.2 since Eq (26) holds; the other case is *g*_*ii*_ = 1 (*r*_*ii*_ = 0 at this time), combining with Eq (26), one can see that the contradiction appears according to the rule 1.5. In summary, if $S_{i}^{0}\left(t+1\right)=0$, it is also impossible that *S*^0^(*t*) obeys all the rules and dissatisfies Eq (18) at the same time.

These results demonstrate that the state *S*^0^(*t*), which obeys all the rules but dissatisfies the Eq (18), does not exist. Therefore, *U* is a subset of *V* (*U* ⊂ *V*), and *U* = *V* is proved. Now, we can conclude that the states found with our algorithm are precisely all the pre-images of the target network state.

**Fig 6:**
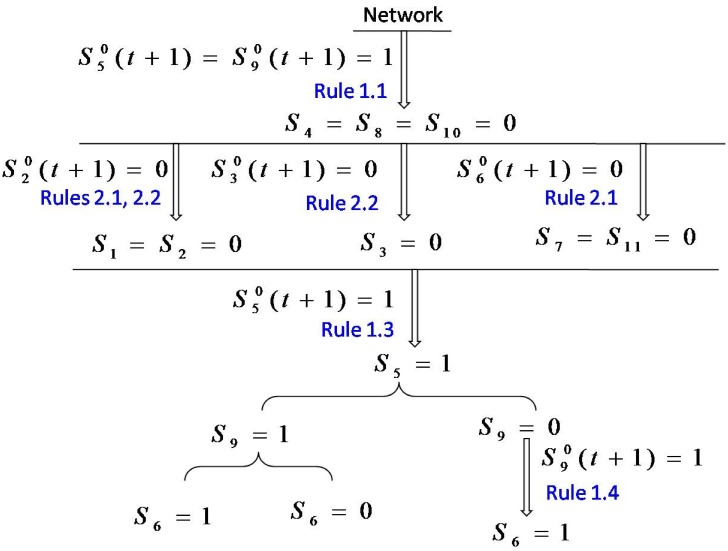
The flow chart for determing the pre-images of target state *S* = (0, 0, 0, 0, 1, 0, 0, 0, 1, 0, 0).

As an example, we will show that how we find the pre-images of one singleton attractor (0, 0, 0, 0, 1, 0, 0, 0, 1, 0, 0) of the cell-cycle network of budding yeast. For this case, *S*^0^(*t* + 1) = (0, 0, 0, 0, 1, 0, 0, 0, 1, 0, 0) in Eq (18). As shown in Fig 6, according to the known network structure and the rules 1.1 − 1.5 and 2.1 − 2.5, the states of many nodes are determined: we obtain *S*_4_ = *S*_8_ = *S*_10_ = 0 based on $S_{5}^{0}\left(t+1\right)=S_{9}^{0}\left(t+1\right)=1$ and the rule 1.1; then, *S*_1_ = 0 and *S*_2_ = 0 are gotten according to $S_{2}^{0}\left(t+1\right)=0$ and the rules 2.1 and 2.2, respectively; according to $S_{3}^{0}\left(t+1\right)=0$ and the observation 2.2, we obtain *S*_3_ = 0; according to $S_{6}^{0}\left(t+1\right)=0$ and the observation 2.1, we obtain *S*_7_ = *S*_11_ = 0; then again according to $S_{5}^{0}\left(t+1\right)=1$ and the rule 1.3, we obtain *S*_5_ = 1. Now, the remain nodes whose states can not be determined are nodes 6 and 9, so we remember the state *S* = (0, 0, 0, 0, 1, *S*_6_, 0, 0, *S*_9_, 0, 0) with *l*_0_ = 2.

For the network state *S* = (0, 0, 0, 0, 1, *S*_6_, 0, 0, *S*_9_, 0, 0) with *l*_0_ = 2. We assign *S*_9_ = 1 firstly. However, the state of node 6 can not be determined, and we find that *S*_6_ = 0 and *S*_6_ = 1 both are the solutions of the equations. So we return the pre-images *S* = (0, 0, 0, 0, 1, 1, 0, 0, 1, 0, 0) and (0, 0, 0, 0, 1, 0, 0, 0, 1, 0, 0). Then, we assign *S*_9_ = 0. According to the rule 1.4, we obtain *S*_6_ = 1 and the pre-image is *S* = (0, 0, 0, 0, 1, 1, 0, 0, 0, 0, 0). Finally, we successfully find three pre-images of the target network state *S*^0^(*t* + 1): *S* = (0, 0, 0, 0, 1, 1, 0, 0, 1, 0, 0), (0, 0, 0, 0, 1, 0, 0, 0, 1, 0, 0) and (0, 0, 0, 0, 1, 1, 0, 0, 0, 0, 0).

**Fig 7:**
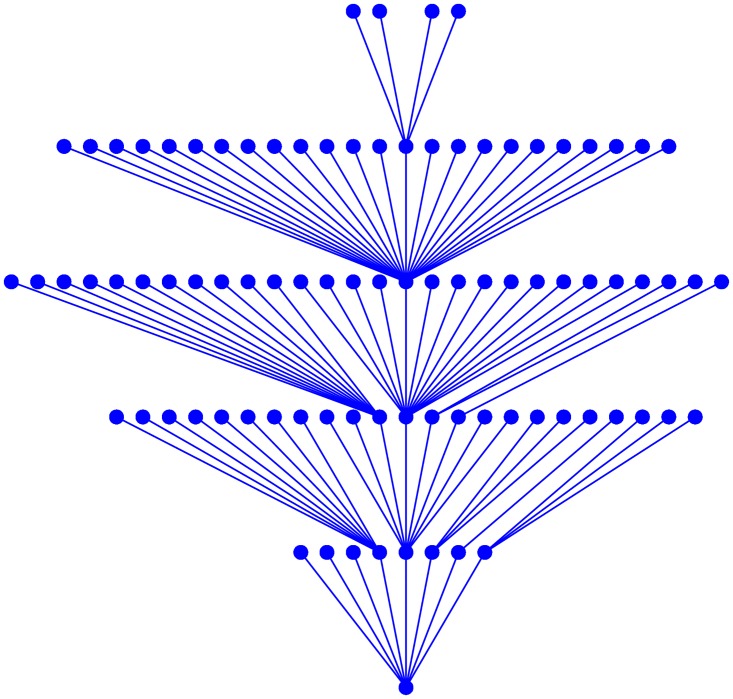
The basin of the singleton attractor (0, 0, 1, 1, 0, 0, 0, 0, 0, 0, 0) is displayed hierarchically.

We can also find the basin of any singleton attractor with this algorithm by reversely inferring the pre-images step by step: we can obtain the pre-images of the singleton attractor first, then find the pre-images of all these known pre-images, go on until all states of the basin are found. The basin of the singleton attractor (0, 0, 1, 1, 0, 0, 0, 0, 0, 0, 0) is displayed hierarchically in Fig 7, where we can clearly see how far a state is from the singleton attractor.

## Conclusion

In summary, we have presented a novel algorithm for finding the singleton attractors in strong-inhibition Boolean networks. The average case time complexity of the proposed algorithm is approximately *O*(1.34^*N*^) which is much faster than the enumeration-based algorithm. It may not be faster than the out-degree based ordering algorithm in [30] for networks with very low maximum indegree. However, we find that the computational time assumed of the algorithm is proportional to the number of the singleton attractors, it shows that our algorithm will work much better for networks with less singleton attractors, especially for the networks with high average degree and less inhibitory interactions. What’s more, the algorithm can be extended to identify the pre-images of any network state and the basin of any singleton attractor. Therefore, the proposed algorithm could be effective and practical. We hope it has good applications in the study of biological networks. On the other hand, we can also know that the algorithm has its own limitations. For example, our algorithm completely relies on the strong-inhibition Boolean model, it may not be workable for other kinds of model, such as the general Boolean model. The computational time of the algorithm increases exponentially with the size of network, so it may not be applied to networks with several hundreds or more nodes. Moreover, we didn’t get the theoretical results of the time complexity of the algorithm though we have made great efforts. We hope some of these limitations can be overcome and this work can be further improved in the future.

## Supporting Information

S1 File Data and code of the algorithm. This data set contains the code of the algorithm for finding the singleton attractors and pre-images in strong-inhibition Boolean networks. The adjacency matrices of five classical networks are also given.
(ZIP)
